# Multimodal Ensemble Deep Learning to Predict Disruptive Behavior Disorders in Children

**DOI:** 10.3389/fninf.2021.742807

**Published:** 2021-11-24

**Authors:** Sreevalsan S. Menon, K. Krishnamurthy

**Affiliations:** Department of Mechanical and Aerospace Engineering, Missouri University of Science and Technology, Rolla, MO, United States

**Keywords:** deep learning, disruptive behavior disorders, multimodal ensemble learning, neuroimaging, 3D CNN

## Abstract

Oppositional defiant disorder and conduct disorder, collectively referred to as disruptive behavior disorders (DBDs), are prevalent psychiatric disorders in children. Early diagnosis of DBDs is crucial because they can increase the risks of other mental health and substance use disorders without appropriate psychosocial interventions and treatment. However, diagnosing DBDs is challenging as they are often comorbid with other disorders, such as attention-deficit/hyperactivity disorder, anxiety, and depression. In this study, a multimodal ensemble three-dimensional convolutional neural network (3D CNN) deep learning model was used to classify children with DBDs and typically developing children. The study participants included 419 females and 681 males, aged 108–131 months who were enrolled in the Adolescent Brain Cognitive Development Study. Children were grouped based on the presence of DBDs (*n* = 550) and typically developing (*n* = 550); assessments were based on the scores from the Child Behavior Checklist and on the Schedule for Affective Disorders and Schizophrenia for School-age Children-Present and Lifetime version for DSM-5. The diffusion, structural, and resting-state functional magnetic resonance imaging (rs-fMRI) data were used as input data to the 3D CNN. The model achieved 72% accuracy in classifying children with DBDs with 70% sensitivity, 72% specificity, and an F1-score of 70. In addition, the discriminative power of the classifier was investigated by identifying the cortical and subcortical regions primarily involved in the prediction of DBDs using a gradient-weighted class activation mapping method. The classification results were compared with those obtained using the three neuroimaging modalities individually, and a connectome-based graph CNN and a multi-scale recurrent neural network using only the rs-fMRI data.

## 1. Introduction

Magnetic resonance imaging (MRI) is a powerful noninvasive neuroimaging tool that can reveal anatomical features and neuronal activities inside a brain. MRI data is widely used to study cognitive development, pathologies, and psychiatric disorders. Diffusion MRI (dMRI) can reveal information about the microstructures, fiber connections, and anatomical connectivities within the brain, and the static anatomical images acquired using structural MRI (sMRI) provide information about the gross anatomical structures in the brain. Dynamic activities inside the brain are measured using functional MRI (fMRI), which is used to identify brain activities in the absence of a task (resting-state fMRI; rs-fMRI) or during a task (task fMRI; tfMRI).

Disruptive behavior disorders (DBDs) include oppositional defiant disorder (ODD; a pattern of angry/irritable mood, argumentative/defiant behavior, or vindictiveness lasting at least 6 months) and conduct disorder (CD; behavior in which the basic rights of others or major age-appropriate societal norms or rules are violated; American Psychiatric Association, 2013). They are prevalent in children and the most common reasons for referring children to mental health services (Hawes et al., 2020). ODD is estimated to occur in 2–16% of youth, depending on the population being studied and the method for diagnosis, and CD, which is more prevalent among younger males, rates range from 6 to 9% (SAMHSA, 2011). DBDs are associated with increased risk for other mental health and substance use disorders (Nock et al., 2006), and are predictors of poor mental health conditions (Scarmeas et al., 2007). These disorders can cause substantial economic losses for society in terms of service utilization (Rivenbark et al., 2018). Therefore, early diagnosis of DBDs is crucial to lower the risk for subsequent disorders with appropriate psychosocial interventions and treatment. However, DBDs are challenging to diagnose as they are often comorbid with other disorders, such as attention-deficit/hyperactivity disorder, anxiety, and depression (Allen et al., 2020).

Machine learning concepts are now receiving increased attention for analysis and prediction in neuroimaging applications. Traditional machine learning techniques require hand-engineered feature selection, which are time-consuming and prone to bias due to manual feature selection. Deep learning is a recent development in machine learning that overcomes the issues associated with hand-engineering and requisite domain expertise for feature selection. Deep learning is a representation learning in which raw data are fed into a learning algorithm that decomposes it into multiple levels of complex nonlinear representative patterns of the input data (LeCun et al., 2015). The burgeoning wide applications of deep learning models can be attributed to the implementation of a convolutional neural network (CNN) because it cut the second-best error rate for image classification by nearly half at the ImageNet Large-Scale Visual Recognition Challenge in 2012 (Krizhevsky et al., 2012). With the advent of parallel computing and graphics processing units, deep representation learning was successfully implemented in numerous areas, such as image processing and analysis tasks, natural language processing, speech recognition, and data synthesis and analysis (LeCun et al., 2015). A large number of medical image analyses now focus on applying deep learning methods to extract features from raw data for further analysis and interpretation (Lundervold and Lundervold, 2019).

CNNs inspired by visual neuroscience are one of the widely used deep learning architectures. A typical CNN includes a convolutional layer, pooling layer, and fully connected layer. The convolutional layer consists of filters/kernels of fixed size that strides with a partial overlap through the input and generates feature maps that are locally weighted sum of input features. Each filter in a convolutional layer looks for the same pattern in different parts of the input, and outputs a unique feature map. The convolution filter thus looks for highly correlated local motifs that can occur at any location in the input (LeCun et al., 2015). The feature maps in the convolution layer are then passed through nonlinear activation functions, such as the rectified linear unit (ReLu) (O'Shea and Hoydis, 2017). The output from one or more convolution layers is then pooled in a pooling layer that merges similar features. Pooling filters output the average or maximum value inside the filter grid and impart translational invariance, for inputs with minor shifts and distortions in rows or columns, to the activation map. Typically, several convolutional and pooling layers are stacked in a CNN, and they are followed by a fully connected layer. The fully connected layer usually connects to an output layer, which could be a softmax function for classification tasks or a linear or support vector machine for regression tasks. CNNs learn in a hierarchical fashion from low-level features, such as edges (similar to primary visual cortex), to high-level features, such as shapes (identical to the secondary visual cortex), in deep layers similar to the hierarchical structure in a human visual cortex (Hubel and Wiesel, 1962). Brainnet CNN (Kawahara et al., 2017) is an earlier developed connectome-based graph CNN which is composed of edge-to-edge, edge-to-node, and node-to-graph convolutional filters that leverage the topological locality of brain networks as opposed to local spatial filtering.

CNNs are often considered “black boxes” that perform classifications without explanations on what a model learned or which part of an input was responsible for the classification. One primary goal of machine learning in neuroimaging is to reveal neuromarkers that are indicative of brain health, and diseases and disorders (Khosla et al., 2019). To address these issues, visualization techniques can be utilized to discover discriminative features learned by a CNN model. Class activation mapping (CAM) is a technique to obtain visual explanations of the input regions that a CNN emphasized in its classification (Zhou et al., 2016; Selvaraju et al., 2017) by calculating the derivative of the CNN classification function estimated via back-propagation with respect to the input data. Gradient CAM (Grad-CAM) and Grad-CAM++ are two improved versions of CAMs because they can be applied to a wide variety of networks without global average pooling and retraining, and they reveal the discriminative regions in any CNN architecture (Selvaraju et al., 2017; Chattopadhyay et al., 2018). The three CAM techniques were compared in one study on classifying multiple sclerosis types, and it was shown that Grad-CAM outperformed CAM and Grad-CAM++ (Zhang et al., 2021).

A multi-scale recurrent neural network (MsRNN) is another deep learning-based framework that can directly work on the dynamic spatiotemporal fluctuations in the brain activity measured using rs-fMRI time courses for identifying brain disorders (Yan et al., 2019). While the CNN models, *deep in space*, can be used as an “encoder” for obtaining correlations between brain regions, recurrent neural network (RNN) models, *deep in time*, can be utilized in sequence classification (Yan et al., 2019). A simple RNN consists of input, hidden and output layers, and it processes the input sequentially with respect to time. The distinguishing feature of RNNs is that the output from a layer is used as input for the layer itself, thereby forming a feedback loop. This allows the RNN to have a history of the sequence elements that can be used to predict the upcoming sequence elements.

Several studies in machine learning showed that the performance of the learning algorithm can be improved using ensemble learning, which is an algorithm-independent machine learning strategy (Opelt et al., 2004; Khosla et al., 2019). Moreover, brain abnormalities are heterogeneous and cause alterations in functional connectivity and structural changes (McLaughlin et al., 2019). Studies have found abnormal brain activities in children with DBDs using dMRI (Hummer et al., 2015), sMRI (Wallace et al., 2014; Hummer et al., 2015; Waller et al., 2020), tfMRI (Rubia et al., 2009; Hawes et al., 2020), and rs-fMRI (Lu et al., 2015; Werhahn et al., 2020). Therefore, there is significant motivation to take advantage of complementary information on various aspects of neuropathology. This study addresses a knowledge gap in the availability of multimodal tools for studying brain abnormalities using different neuroimaging modalities.

In this study, a 3D CNN ensemble deep learning model framework with multimodal neuroimaging data was exploited to identify children with DBDs. The dMRI, sMRI, and rs-fMRI data from a subsample of children enrolled in the Adolescent Brain Cognitive Development (ABCD) Study (Casey et al., 2018) were used as the input data. Furthermore, the brain regions involved in classifying children with DBDs were identified utilizing Grad-CAM that illustrated the discrimination power of the classifier and the ability to identify neuroimaging phenotypes for DBDs. To assess improvements offered by the ensemble learning, the results were compared with those obtained using the three neuroimaging modalities individually; they were also compared with those obtained using two other readily available deep-learning frameworks, Brainnet CNN and an MsRNN, model with rs-fMRI data. We hypothesized that the classification performance of the ensemble deep learning model will be significantly better than the single modality models.

## 2. Materials and Methods

### 2.1. Dataset

Data used in this study came from the ABCD Study that recruited 11,878 children (48% female; 52% male) between 108 and 120 months of age across 21 sites in the United States. A detailed description of the recruitment, demographics, physical health, and mental assessment and imaging protocols for the study can be found elsewhere (Barch et al., 2018; Casey et al., 2018; Garavan et al., 2018). The baseline ABCD Study data used in this study were from the annual 2.0.1 data release and can be downloaded from the National Institute of Mental Health (NIMH) Data Archive^1^. The data is available to qualified researchers at no cost after their NIMH Data Archive Data Use Certification has been approved. Children with DBDs were identified using the Child Behavior Checklist (CBCL) and the Schedule for Affective Disorders and Schizophrenia for School-Age Children-Present and Lifetime version for DSM-5 (K-SADS-PL) (Hawes et al., 2020). Specifically, the criterion included children who: (i) scored at or above the borderline clinical range (i.e., T-scores ≥67) on either the CBCL DSM-oriented conduct problems subscale or oppositional defiant problems subscale; or (ii) received a K-SADS-PL conduct disorder or oppositional defiant disorder diagnosis. Based on this criterion, there were 1,100 children with minimally preprocessed data with all three neuroimaging modalities, i.e., dMRI, sMRI, and rs-fMRI.

### 2.2. Preprocessing of ABCD Study Minimally Preprocessed Data

DTI data were preprocessed using FSL (FMRIB's Software Library^2^) scripts, which were used to perform nonlinear registration and projection onto an alignment-invariant tract representation of fractional anisotropy (FA) and mean diffusivity (MD). First, diffusion tensor models were fit at each voxel by using FMRIB's Diffusion Toolbox (FDT, part of FSL). Second, brain extraction was performed using the brain extraction tool (BET) (Smith, 2002). Third, nonlinear registration was done, thereby aligning all FA and MD images to a FMRIB58_FA standard-space image, which has a 1 × 1 × 1 mm resolution, as the target. Finally, all images were resampled back to the 2 × 2 × 2 mm FSL default MNI152 standard-space template resolution. Figure S1 shows an example DTI image.

The sMRI T1-weighted images were preprocessed mainly using the FSL software. First, extraction of the brain tissue from the skull was performed by using BET. Second, registration to standard space images was carried out using FLIRT (Jenkinson and Smith, 2001; Jenkinson et al., 2002). Third, registration from high-resolution structural to the FSL default MNI152 standard space was then further refined using FNIRT nonlinear registration (Andersson et al., 2007a,b). Finally, the FMRIB's Automated Segmentation Tool (FAST) (Zhang, 2001) was used to segment the brain 3D-image into three different tissue types: (i) gray matter; (ii) white matter; and (iii) cerebrospinal fluid (CSF). Figure S2 shows an example sMRI image.

The rs-fMRI data preprocessing was carried out using FEAT (FMRI Expert Analysis Tool) Version 6.00, a part of FSL. Registration to high-resolution structural and the FSL default MNI152 standard space images was carried out using FLIRT. Registration from high-resolution structural to standard-space was further refined using FNIRT nonlinear registration. Additionally, the following pre-statistics processing was applied: (i) motion correction using MCFLIRT (Jenkinson et al., 2002); (ii) non-brain removal using BET; (iii) spatial smoothing using a Gaussian kernel of FWHM 8.0 mm; (iv) grand-mean intensity normalization of the entire 4D dataset by a single multiplicative factor, which was done by default in all the fMRI software packages to ensure each image scan had roughly the same mean; and (v) high-pass temporal filtering (Gaussian-weighted least-squares straight-line fitting, with sigma = 50.0 s). The Pearson seed-based correlation values were calculated for four regions of interest, namely posterior and anterior cingulate cortex (PCC and ACC), medial prefrontal cortex (mPFC) and ventral caudate, which are known to be affected in children with DBDs (Alegria et al., 2016). Figure S3 shows an example rs-fMRI image for the ACC.

Children were removed from the study pool following preprocessing due to high motion (framewise displacement >0.25 mm), misalignment, and registration failures. As a result, the complete preprocessed data were available for 550 children and a matching number of children in age and sex without DBDs (typically developing, TD) were selected from the ABCD Study data as the control group. Table 1 shows the demographic and clinical characteristics of the final study pool. Descriptive statistics show that the groups were equivalent on demographic variables and significantly different on clinical scores.

**Table 1:** Demographic and clinical characteristics of the study pool.

**Characteristic**	**DBDs**			**TD**			***p*-value**
	**Mean**	**SD**	**%**	**Mean**	**SD**	**%**	
**Demographics**							
Age (months)	118.3	7.7		118.7	7.4		0.38
Sex (male)			61.6			62.2	0.85
Race
African American			16.2			14.0	
Caucasian			54.0			53.8	0.44
Hispanic			16.2			19.5	
Other			13.6			12.7	
**Clinical**							
CBCL CP subscale	63.6	8.13		50	0		<0.001
CBCL ODD subscale	63.9	7.44		50	0		<0.001
KSADS-PL CD diagnosis			29.6			0	<0.001
KSADS-PL ODD diagnosis			73.3			0	<0.001

### 2.3. Ensemble Learning

**Figure 1:**
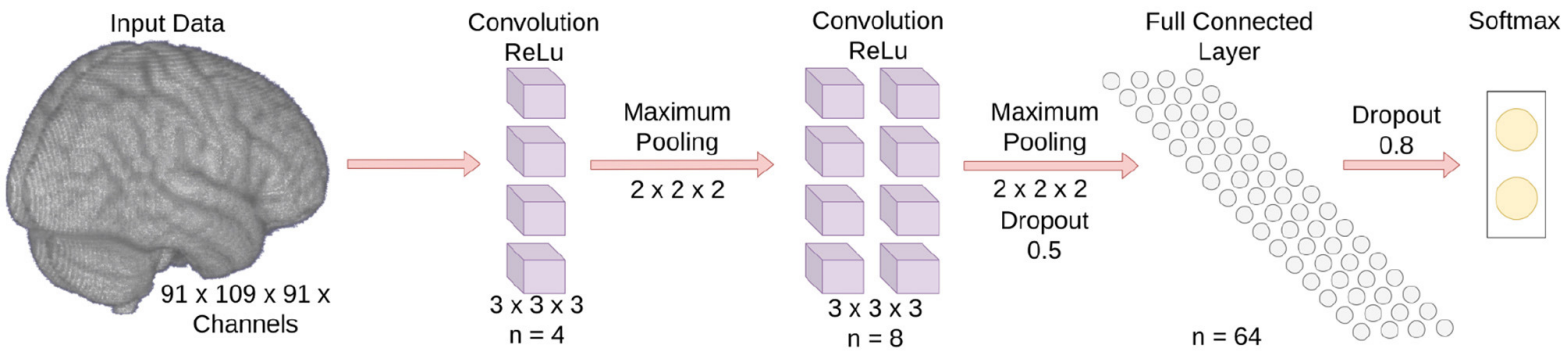
Schematic of the 3D CNN model. The number of input data channels is not shown because it varies depending on the input modality.

Three multichannel 3D CNNs whose inputs were dMRI, sMRI, and rs-fMRI, respectively, were trained in this study to classify children with DBDs and TD children. The goal for the 3D CNNs was to learn the mapping between input (features related to the microstructural integrity and gross anatomical structure of the brain, and resting-state functional patterns) and label (TD children and children with DBDs), so that the 3D CNNs can predict DBDs in previously unseen children. As shown in Figure 1, each 3D CNN model had two convolution blocks each consisting of a 3D convolutional layer (kernel size 3, stride 1), a ReLU activation layer, and a max-pooling layer (kernel size 2, stride 2). The number of feature channels were 4 and 8 for the convolution layers, respectively. The last layer was a fully connected layer with 64 neurons to combine the feature vectors, and a dropout layer was used to reduce model overfitting. The output was a softmax classification layer. The input channels for the three 3D CNN models were as follows: (i) dMRI model—two channels for FA and MD values; (ii) sMRI model—three channels for gray matter, white matter, and CSF; and (iii) rs-fMRI model—four channels for Pearson correlation of seed regions ACC, PCC, mPFC, and ventral caudate. The three models were combined in an ensemble learning strategy that gave equal weight during maximum voting of the softmax output for classifying children with DBDs and TD children.

The 3D CNN models were trained with mini-batch sizes of 32 with early stopping conditioned on validation accuracy. The binary cross-entropy was used as the loss function and the neural network weights were optimized using the Adam optimizer. The learning rate and gradient decay were set to 0.001 and 0.9, respectively. The squared gradient decay, epsilon, and maximum epochs were set to 0.9, 0.001, and 50, respectively. No attempt was made to optimize the aforementioned parameters. To ensure that all 3D CNN models relied on information from the same voxels, the FSL default MNI152 standard-space mask was applied to the voxel-level data before feeding into the 3D CNN model (Khosla et al., 2019). This step removed voxels that may have emerged outside the standard brain template because the preprocessing transformation matrix does not create the exact brain boundary.

### 2.4. Brainnet CNN

**Figure 2:**
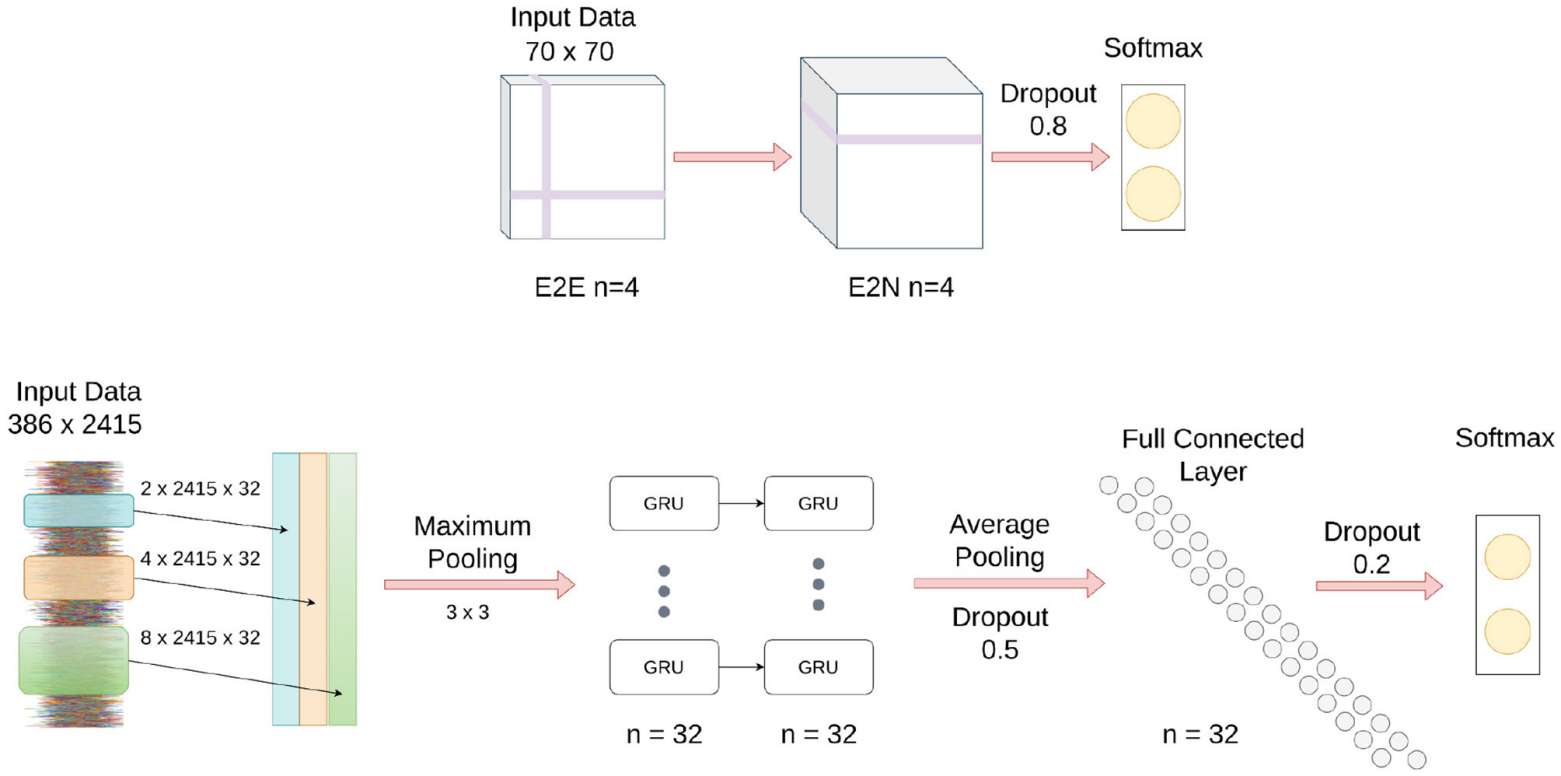
Schematic of Brainnet CNN and MsRNN architecture. **(Top)** Brainnet CNN; the edge to edge (E2E) layer uses a crosshair convolution filter, and the edge to node (E2N) layer uses a 1D convolution row filter. **(Bottom)** MsRNN; three varied convolutions are first performed in the input layer, the output is then concatenated, and finally maximum pooled before being fed into the gated recurrent units (GRUs).

As shown in Figure 2, input to the Brainnet CNN was a functional connectivity matrix obtained using timeseries extracted from 70 resting-state networks, which were identified using publicly available 70-component independent component analysis maps (Smith et al., 2009). The blood oxygenation level-dependent (BOLD) timeseries were extracted from the 70 brain areas by averaging the BOLD signal over all voxels belonging to each brain area. The timeseries were detrended and demeaned, and the data were bandpass filtered in the range of 0.01–0.15 Hz to improve identification of the resting-state fluctuations (Menon and Krishnamurthy, 2019a). The functional connectivity matrix was obtained using Pearson correlation with normalization to z-scores using the Fisher transformation.

The Brainnet CNN model was implemented in Python by modifying publicly available scripts (Kawahara et al., 2017). The Brainnet CNN model had an edge-to-edge (E2E) layer with four filters, followed by a edge-to-node (E2N) layer with four filters, and finally a dense layer with two neurons. A leaky ReLU non-linearity with alpha equal to 0.33 was applied to the output of each layer except the last layer, which was a softmax layer. Dropout regularization with a rate of 0.8 was used for the edge-to-node layer and cross-entropy loss was used to optimize the classification model. The models were trained for 1,000 iterations using stochastic gradient descent with a momentum equal to 0.9. The learning rate was set to 0.001 and a decay of 0.0005 was used for the classification model. No attempt was made to optimize the aforementioned parameters.

### 2.5. Multi-Scale Recurrent Neural Network

Figure 2 shows a schematic of the MsRNN used in this study. The timeseries extracted from 70 resting-state networks that were input into the Brainnet CNN were also used as the input to an MsRNN. The dynamic correlation connectivity values of 2,415 edges were calculated with a window length of 85 TR and step size of 5 TR (Menon and Krishnamurthy, 2019a). The MsRNN utilized three different scales of 32 1D convolutional filters (2 TR, 4 TR, and 8 TR, TR = 0.8 s), one concatenation layer, one max-pooling layer of kernel size 3, a two-layer stacked gated recurrent unit GRU with 32 filters which were densely connected in a feed-forward manner, and an averaged layer that integrated the whole sequence followed by a dense layer of 32 neurons before the softmax classification layer. Dropout layers were used before and after the dense neurons with 50 and 20% dropout, respectively, and L1 and L2 regularization of 0.01 was used to avoid overfitting the data. The MsRNN was trained in Python following Yan et al. (2019) with a mini-batch size of 32, and included early stopping conditioned on validation accuracy and a learning rate of 0.001. The binary cross-entropy was used as the loss function, and the neural network weights were optimized using the Adam optimizer. No attempt was made to optimize the aforementioned parameters.

## 3. Results

### 3.1. Experiments

To test the efficacy of the multimodal data ensemble, a ten-fold cross-validation (CV) strategy with maximum voting was investigated. The Grad-CAM method was applied to the predicted output, and the results for all the children with DBDs and TD children were averaged to delineate the global trends of the important regions involved in the classification. To benchmark the performance of the ensemble learning approach, the results were compared to those obtained from: (i) the three 3D CNN models used in the ensemble learning considered individually; (ii) Brainnet CNN; and (iii) MsRNN model.

### 3.2. Classification Performance

**Figure 3:**
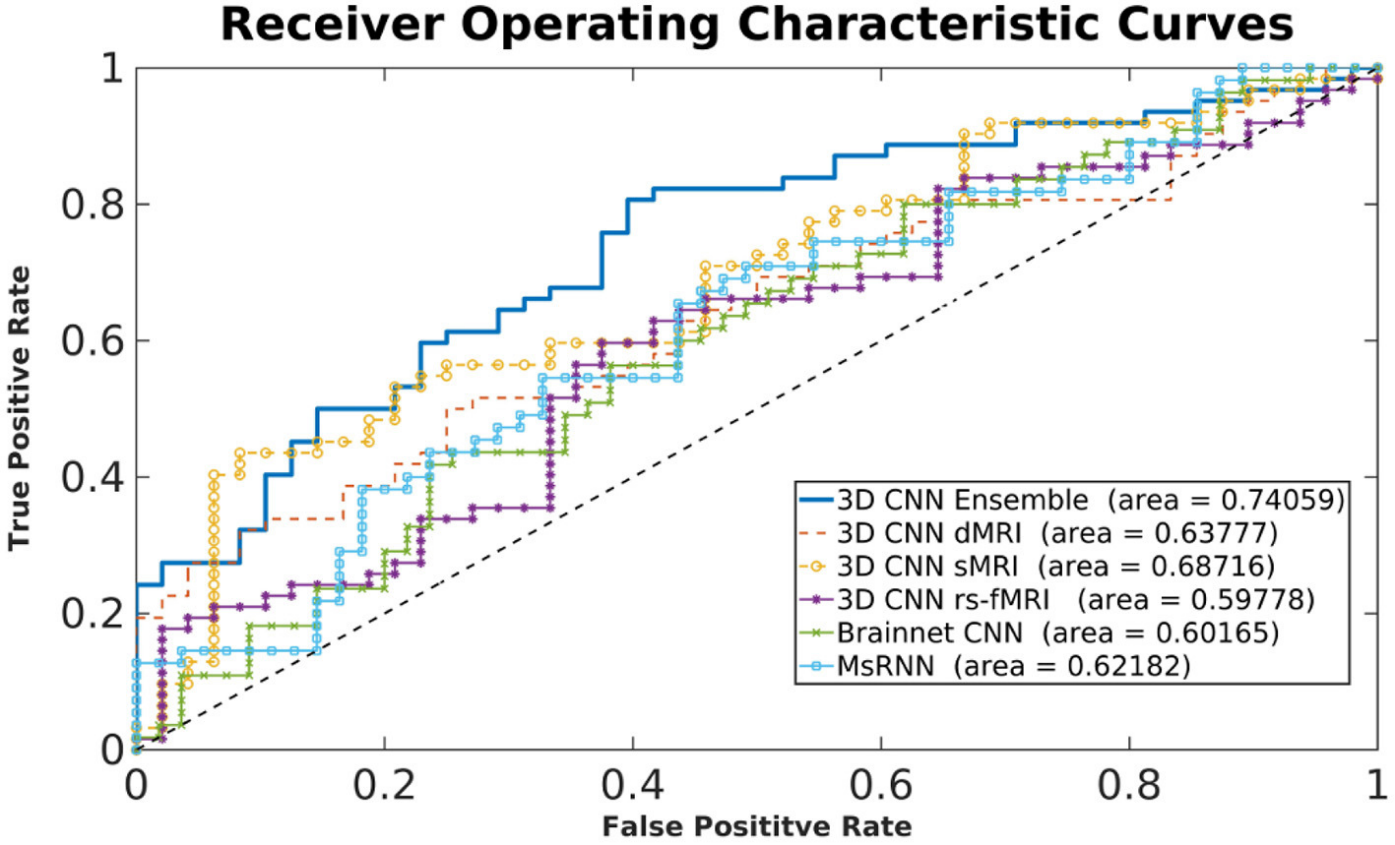
Typical receiver operating characteristic curves for different models.

Figure 3 shows typical receiver operating characteristic curves and Table 2 shows the performance of the different methods for classifying children with DBDs and TD controls. With 10-fold cross-validation, the multimodal ensemble model with maximum voting resulted in an average prediction accuracy to 72%. The average prediction accuracies for dMRI, sMRI, and rs-fMRI single modalities were 64, 66, and 66%, respectively, compared to 62% with the Brainnet CNN and MsRNN. Table 2 also shows the multimodal ensemble model to have higher sensitivity, specificity, and F1-score compared to the other models considered.

**Table 2:** Classification performance in percentage.

**Method**	**Modality**	**Accuracy**			**Sensitivity**	**Specificity**	**F1-score**
		**Mean (SD)**	***p*-value**	**Cohen's *d***	**Mean (SD)**	**Mean (SD)**	**Mean (SD)**
3D CNN Ensemble		72 (4.5)	Proposed model		70 (17.0)	72 (15.6)	70 (9.0)
	dMRI	64 (2.6)	<0.001	2.20	60 (16.0)	67 (14.3)	61 (9.7)
3D CNN	sMRI	66 (2.2)	<0.001	1.85	64 (11.2)	65 (13.2)	64 (6.4)
	rs-fMRI	66 (3.0)	0.002	1.57	62 (15.4)	69 (16.4)	64 (7.2)
BrainnetCNN	rs-fMRI	62 (2.9)	<0.001	2.67	60 (7.3)	64 (4.3)	61 (4.5)
MsRNN	rs-fMRI	62 (2.5)	<0.001	2.79	56 (7.7)	68 (8.0)	59 (4.4)

Statistical results from two-sample *t*-tests were used to compare the accuracy of the classification performance of the different models. The higher accuracy of the proposed ensemble model compared to all the other models was significant (highest *p*-value was 0.002) with a very large to huge effect size calculated as Cohen's *d* (Sawilowsky, 2009). Overall, as hypothesized, the classification performance was significantly higher using the ensemble learning model because it utilized complementary information from the three different modalities. The results also indicated the superiority of voxel-based 3D CNN models compared to network-level models, such as Brainnet CNN and MsRNN.

### 3.3. Visualization

**Figure 4:**
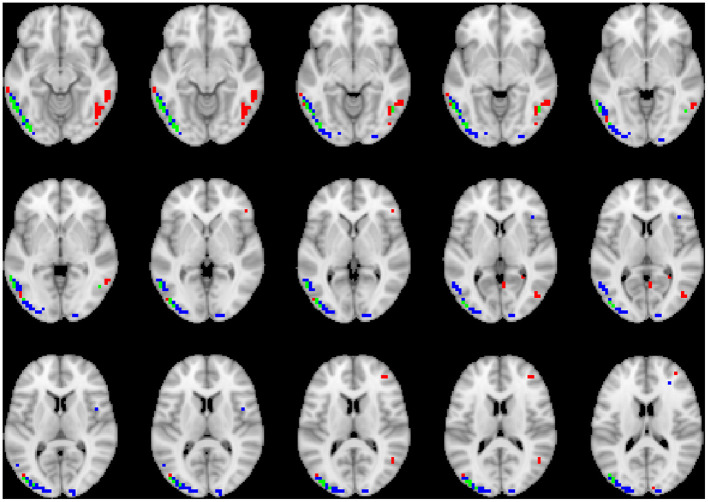
Axial views of voxels primarily contributing to children classification in dMRI image. Green, common to DBD and TD groups; red, DBD group; blue, TD group. The right side of each image corresponds with the right side of the brain.

**Figure 5:**
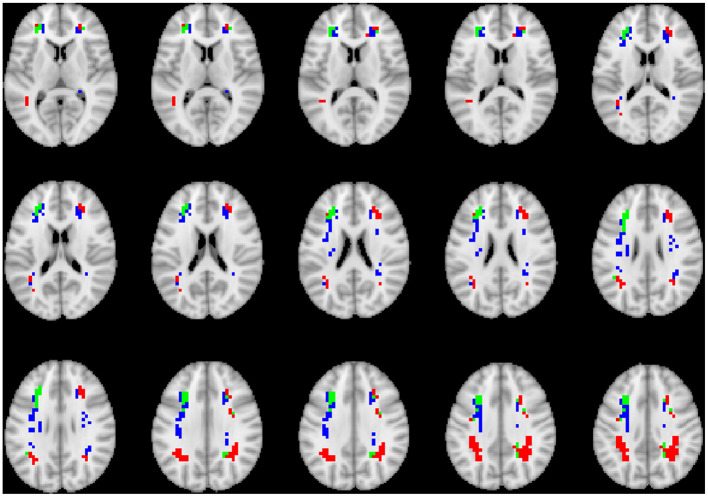
Axial views of voxels primarily contributing to children classification in sMRI image. Green, common to DBD and TD groups; red, DBD group; blue, TD group. The right side of each image corresponds with the right side of the brain.

**Figure 6:**
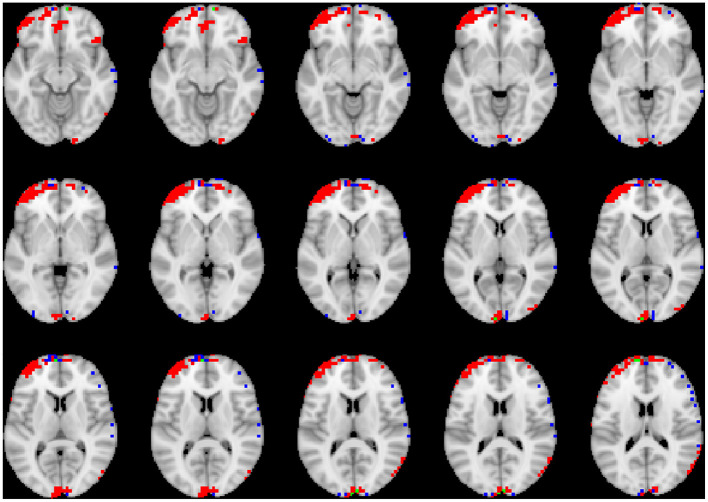
Axial views of voxels primarily contributing to children classification in rs-fMRI image. Green, common to DBD and TD groups; red, DBD group; blue, TD group. The right side of each image corresponds with the right side of the brain.

To visualize the brain regions that primarily contributed to children classification, Grad-CAM obtained for children with DBDs and TD children were thresholded at 99 percentile to first identify voxels with high gradient values. The brain regions that primarily contributed toward classification were then identified using the JHU ICBM-DTI-81 white-matter atlas for dMRI image and the AAL atlas for sMRI and rs-fMRI images. Figures 4–6 show axial views of voxels that primarily contributed to the classification of children with DBDs and TD children in dMRI, sMRI, and rs-fMRI images, respectively. Table 3 lists the top five brain regions in the dMRI images and top ten in the sMRI and rs-fMRI images of children with DBDs and TD children. Some of the regions were common to both groups and are listed in green. On the other hand, regions that were unique to children with DBDs and TD children are listed in red and blue, respectively. These unique regions are of interest because their contributions outweigh the gradient contributions of common regions, hence are highly class discriminative. Further, these unique regions are evidence of abnormalities in children with DBDs because the primarily contributing regions are all different from those in the TD children.

**Table 3:**
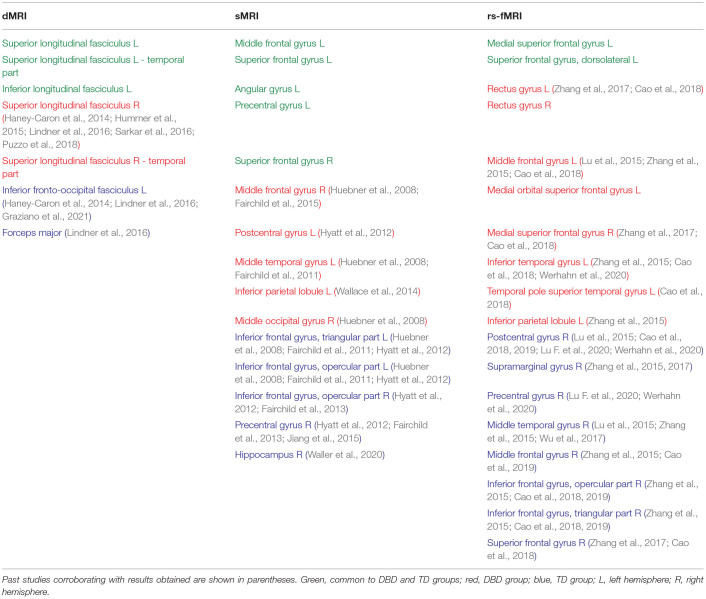
Brain regions primarily contributing to children classification.

### 3.4. 3D CNN Training Information

Time for training the 3D CNN model was similar for the three modalities. It typically took around 150 min per fold on Dell Precision 7,910 and 7,920 Tower workstations with Intel Xeon processors, 128 GB RAM and 1 GB GPU. Figures S4, S5 show a typical training graph and Precision-Recall curves, respectively.

## 4. Discussion

This was the first neuroimaging study to consider the classification of children with DBDs. This is a challenging problem because DBDs are often comorbid with other disorders, such as attention-deficit/hyperactivity disorder, anxiety, and depression. The multimodal ensemble learning approach for diagnosing DBDs with voxel-based 3D CNN is a novel approach and the accuracy of the ensemble model increased by 6–10% compared to other models. The maximum voting in the ensemble learning method simulates how clinicians typically make decisions. Given that brain abnormalities are heterogeneous, it is naturally advantageous to utilize information from multimodalities. The maximum voting is the simplest and easiest ensemble method that can be applied to 3D CNN models. The maximum voting strategy also ensures that the results are not biased toward any single modality, but will take into account all available information. 3D CNN models, unlike traditional machine learning methods, such as artificial neural networks or support vector machines are well suited to include the spatial relations in the 3D neuroimaging data, which are known to affect brain functioning. Furthermore, traditional machine learning methods will overfit the data and reduce the validation classification accuracy with high-dimensional 3D neuroimaging training data.

Grad-CAM reveals the discriminant regions in the brain that contributed to the classification of children with DBDs. As shown in Table 3, most of these regions corroborate with results from past studies on abnormal development in children with DBDs. To mention a few, alterations in the white matter integrity of the left inferior fronto-occipital fasciculus were suggested as a potential biomarker of conduct disorder (Graziano et al., 2021). Similarly, superior longitudinal fasciculus areas were shown to have differences in diffusion measurements that suggested poor maturation of structural connections (Hummer et al., 2015) in children with DBDs. Morphological aberrance of frontoparietal and temporal gyrus areas can lead to disruptive behavior (Huebner et al., 2008; Hyatt et al., 2012; Fairchild et al., 2015) and most of these regions were found to be class discriminative in this study. Functional connectivity alterations have been reported for children with DBDs, and class discriminative regions found using grad-CAM were consistent with many of the reported regions (Lu et al., 2015; Werhahn et al., 2020). Functional connectivity values for higher-order cognitive functional regions such as the middle frontal gyrus and superior frontal gyrus were also found to be class discriminative (Lu et al., 2015).

The 72% average accuracy obtained using the ensemble learning approach is good. Because there are no other studies on classifying children with DBDs to benchmark against, some representative neuroimaging studies using deep learning were reviewed to qualify the multimodal ensemble model performance. El Gazzar et al. (2019) trained a 1D-CNN on a publicly available autism dataset with nearly 2000 participants to classify rs-fMRI images with an accuracy of ~65%. The accuracy improved to 66% with a 3D CNN (Thomas et al., 2020). Lu H. et al. (2020) obtained an accuracy of 61% by applying multi-kernel fuzzy clustering based on an auto-encoder to classify participants with autism spectrum disorder (ASD) using the Autism Brain Imaging Data Exchange (ABIDE) database (nearly 1,050 participants). Using an ensemble approach on ABIDE data, a classification accuracy of 72.3% was obtained by Khosla et al. (2019). Similar to DBDs, classification of ASD using machine learning methods is also considered challenging because it varies from person-to-person in severity and combination of symptoms. Other studies with a classification accuracy >70% are typically in cases where the sample size is <200 (see Vieira et al., 2017; Zhang et al., 2020 for an overview). The sample size is an important parameter to consider because a negative relationship between accuracy and sample size has been noted (Pulini et al., 2019).

## 5. Limitations and Future Directions

The robustness of the training models could not be determined by using a leave-site-out cross-validation scheme for the ABCD Study data that was collected from 21 sites with optimized and harmonized measures and procedures (Casey et al., 2018). A k-fold cross-validation was used instead because the number of children from each site in the study pool was imbalanced. The number of children with DBDs varied among the different sites, from a low of 3 to a maximum of 113.

This study investigated the superiority of ensemble learning for classifying brain disorders. The sample size used here was relatively large compared to published works in the field, but it was probably not large enough to take full advantage of CNN models. The models used in this research employed a small number of filters with a shallow architecture, and this decreased the deep learning “black box” depth and not fully fit the training data; and it reduced the computational burden, which is advantageous. A wide range of choices were available to increase the depth of the CNN architecture and optimize the training parameters. Hyperparameter optimization of the CNN architecture and training parameters were not performed because the focus here was to investigate the superiority of multimodal ensemble learning with simple models. Tuning the hyperparameters using a grid or random search method, for example, is computationally intense. A number of different optimization algorithms have been proposed (Yu and Zhu, 2020); developing an efficient scheme to optimize the hyperparameters is a topic for future investigation.

For the Brainnet CNN and MsRNN, there are unexplored options for selecting an atlas. In this study, a commonly used functional atlas was considered with few filters similar to the multimodal CNN. Correlation does not account for higher-order interactions because it is a first-order transformation (El Gazzar et al., 2019); therefore, different voxel measurements for rs-fMRI, such as entropy (Menon and Krishnamurthy, 2019b) and other connectivity measures can be investigated. The dynamic nature of the functional connectivity was not analyzed due to the increased computational requirements. Also, no comparison was performed with linear models because a voxel-wise analysis of linear models would suffer from the issues of high dimensionality.

Two strategies that may deserve attention are transfer learning and data augmentation (Vieira et al., 2017; Zhang et al., 2020). Transfer learning involves applying features learned from one dataset to tune another similar dataset. Gong et al. (2021) successfully applied transfer learning strategy exploiting big data from UK Biobank (Miller et al., 2016) in the Predictive Analysis Challenge 2019 dataset, achieving first place. Data augmentation is a strategy used in computer vision applications to enlarge the sample size by applying transformations to the data. Data augmentation methods are only now being addressed for medical imaging classification tasks, but further studies are needed for investigating disorders using 3D brain images with voxel-level data (Zhang et al., 2020).

## 6. Conclusion

The recent availability of public neuroimaging data, such as the ABCD Study, UK Biobank (Miller et al., 2016), and Child Mind Institute-Healthy Brain Network (Alexander et al., 2017), help researchers to develop novel machine learning techniques for studying brain diseases and disorders. The ensemble method with multiple modalities is ideally suited to model heterogeneity that is typical with brain abnormalities. 3D CNN together with visualization using grad-CAM is a promising way to identify neuroimaging phenotypes for the diagnosis of DBDs. Future studies are needed to investigate the use of other neuroimaging modalities to better understand the pathophysiology of brain disorders.

## Data Availability Statement

Publicly available datasets were analyzed in this study. This data can be found at: Adolescent Brain Cognitive Development Study (https://dx.doi.org/10.15154/1504041). Custom in-house MATLAB and Python scripts are made publicly available in GitHub (https://github.com/sreevalsansmenon/Multimodal_Ensemble).

## Author Contributions

SM and KK: conceptualization, methodology, formal analysis, writing–original draft preparation, and writing–review and editing. SM: software. Both authors contributed to the article and approved the submitted version.

## Author Disclaimer

This manuscript reflects the views of the authors and may not reflect the opinions or views of the NIH or ABCD consortium investigators.

## Conflict of Interest

The authors declare that the research was conducted in the absence of any commercial or financial relationships that could be construed as a potential conflict of interest.

## Publisher's Note

All claims expressed in this article are solely those of the authors and do not necessarily represent those of their affiliated organizations, or those of the publisher, the editors and the reviewers. Any product that may be evaluated in this article, or claim that may be made by its manufacturer, is not guaranteed or endorsed by the publisher.

## Footnotes

^1^ https://dx.doi.org/10.15154/1504041

^2^ www.fmrib.ox.ac.uk/fsl
