# Supplementary material for: Multimodal Ensemble Deep Learning to Predict Disruptive Behavior Disorders in Children
Source: Front Neuroinform. 2021 Nov 24;15:742807. doi: 10.3389/fninf.2021.742807 (PMC8652047; doi:10.3389/fninf.2021.742807)
Supplement: Supplementary file 1 [file Data_Sheet_1.PDF]

Supplementary Material

SUPPLEMENTARY FIGURES

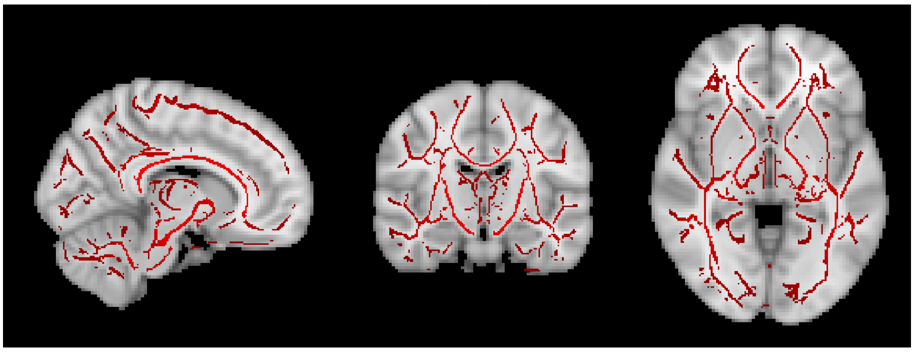

Figure S1. FMRIB58_FA standard-space image (red) overlaid onto the MNI152 standard-space image. The FA and MD values were mapped to the standard-space white matter fiber locations shown in red.

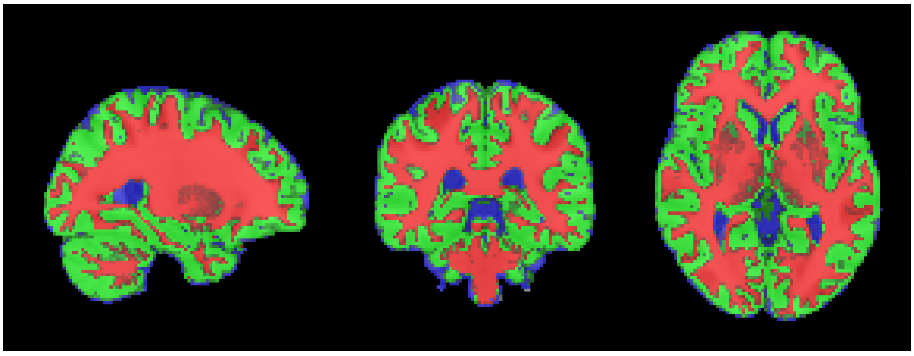

Figure S2. Structural image segmentation showing probability of tissue types in voxels. Red color - white matter, green color - gray matter, and blue color - cerebrospinal fluid. The opacity of each channel was set to 0.5, and probability was thresholded at 0.5 for better contrast in FSLeyes.

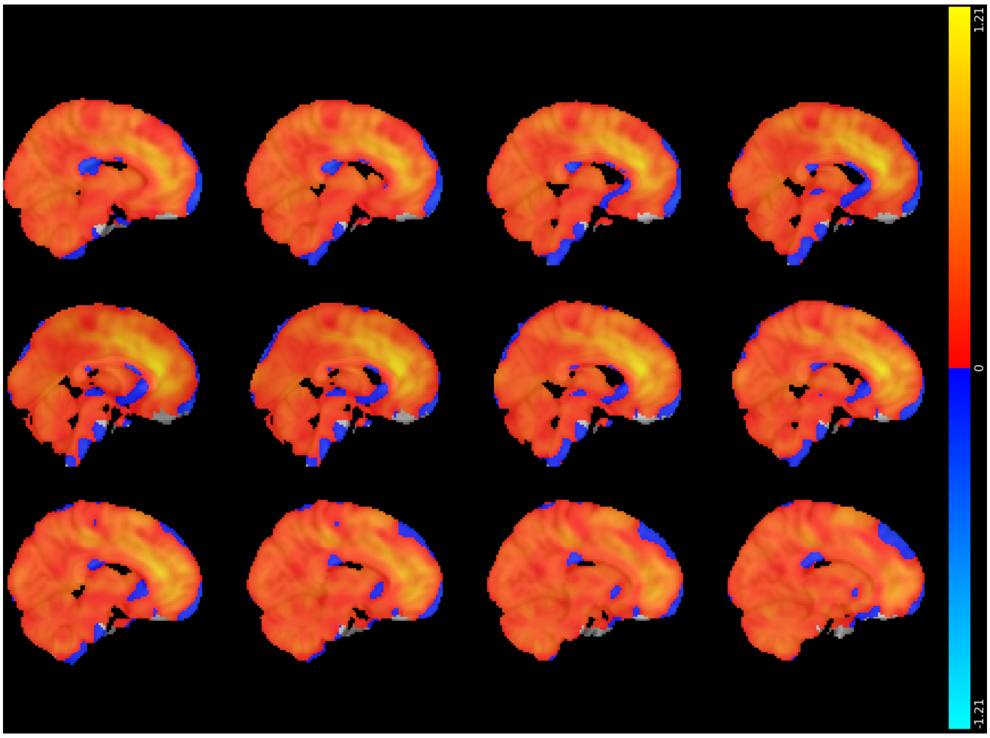

Figure S3. Sagittal views of the brain showing Pearson resting-state seed-based correlation values for anterior cingulate cortex. The color bar to the right of the figure shows the correlation values.

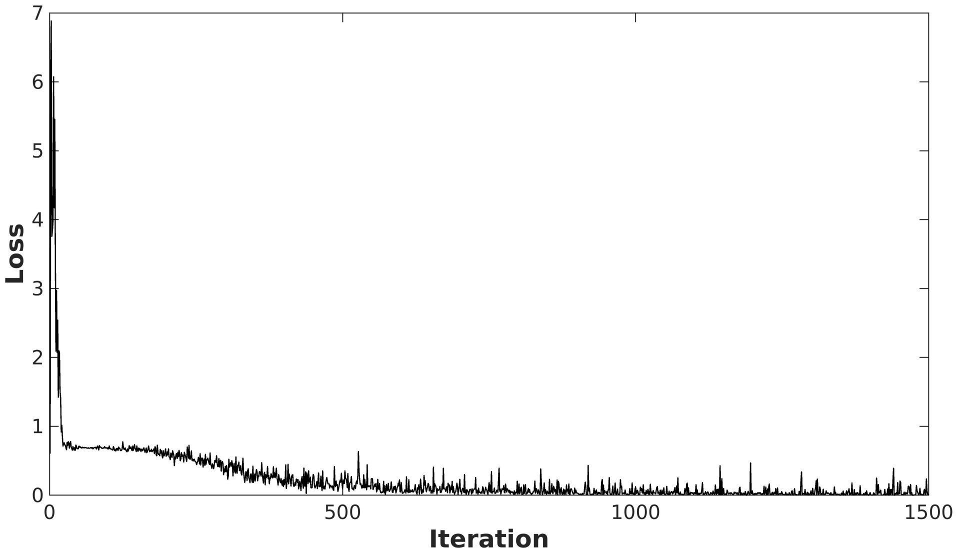

Figure S4. A typical training curve for the 3D CNN rs-fMRI model for 50 epochs.

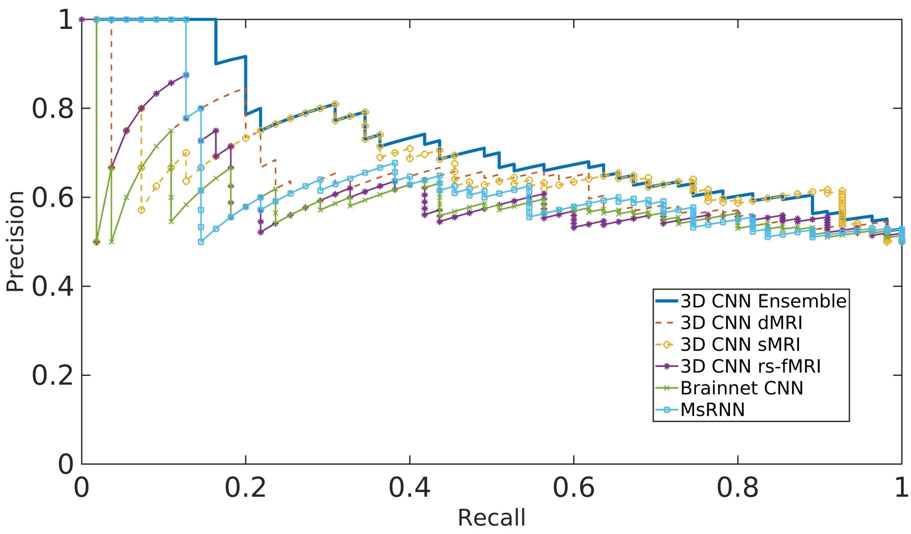

Figure S5. Precision-Recall curves for the different methods for classifying children with DBDs and TD controls.
